# *HCK* can serve as novel prognostic biomarker and therapeutic target for Breast Cancer patients

**DOI:** 10.7150/ijms.43161

**Published:** 2020-09-30

**Authors:** Xudong Zhu, Yixiao Zhang, Yang Bai, Xi Gu, Guanglei Chen, Lisha Sun, Yulun Wang, Xinbo Qiao, Qingtian Ma, Tong Zhu, Jiawen Bu, Jinqi Xue, Caigang Liu

**Affiliations:** 1 Department of Oncology, Shengjing Hospital of China Medical University, Shenyang, Liaoning Province, 110004, China.; 2 Department of Operating Room, Shengjing Hospital of China Medical University, Shenyang, Liaoning Province, 110004, China.

**Keywords:** breast cancer, HCK, prognosis, biomarker, bioinformatic

## Abstract

The role of HCK expression in the prognosis of breast cancer patients is unclear. Thus, this study aimed to explore the clinical implications of HCK expression in breast cancer. We assessed *HCK* expression and genetic variations in breast cancer using Oncomine, GEPIA, UALCAN, and cBioPortal databases. Then, immunochemistry was used to analyze HCK expression in breast cancer specimens, non-cancer tissues and metastatic cancer tissues. Consequently, we evaluated the effect of HCK expression on survival outcomes set as disease-free survival (DFS) and overall survival (OS). Finally, STRING, Coexpedia, and TISIDB database were explored to identify the molecular functions and regulation pathways of HCK. We found that breast cancer tissues have more *HCK* mRNA transcripts than non-cancer tissues. Patients with HCK expression had significantly shorter DFS and OS. The ratio of HCK expression was higher in cancer tissues than in non-cancer tissues. These results from STRING database, FunRich software, and TISIDB database showed that HCK was involved in mediating multiple biological processes including immune response-regulating signaling pathway, cell growth and maintenance through multiple signaling pathways including epithelial to mesenchymal transition, PI3K/AKT signaling pathway, and focal adhesion. Overall, *HCK* may be an oncogene in the development of breast cancer and thus may as a novel biomarker and therapeutic target for breast cancer.

## Introduction

Breast cancer has been the second cause of cancer-related death among women 1. In America, approximately 230,000 women are diagnosed with breast cancer annually 2. Although the identification of the molecular types and the improvement of standard therapy specific to breast cancer types have ameliorated patients' survival outcomes 3,4, effective treatment strategies are yet to be developed in some types of breast cancer patients, such as triple-negative breast cancer. Further, the incidence of both posttreatment recurrence and distant organ metastasis and breast cancer-related death remains high 5-7. Therefore, identifying new biomarkers and potential therapeutic targets may provide new treatment pathways to improve outcomes and prognosis in breast cancer.

Hematopoietic cell kinase (HCK), which belongs to the SRC family of non-receptor protein tyrosine kinases (SFK), is primarily expressed in B lymphocyte lineages and cells of myeloid and is the most abundantly expressed SFKs in the tumor-associated host stroma 8,9. HCK comprises the p61HCK and p59HCK isoforms in human 10,11. Previous researchers found that HCK was involved in innate immune reaction 8,12,13. Further, truncation or phenylalanine missense mutation of the negative regulatory tyrosine residue located in the C-terminal (Y499 in mouse HCK) of HCK lead to aberrant activation of HCK, indicating that HCK may have oncogenic activity 14. Further studies found somatic truncation mutations of *HCK* in 12% patients of colorectal cancer (CRC) 15. Increased HCK expression was also found in pancreatic cancer, CRC, gastric cancer, and other solid malignancies 16-18. The overexpression of HCK may be involved in tumorigenesis, cancer progression, and survival outcomes 19. Accordingly, HCK inhibitors that can suppress pancreatic cancer growth in a preclinical model have been reported 20. These findings predicted that HCK may promote the development and regulation of biological behaviors of cancer.

However, very few studies reported the clinical implications of HCK expression in breast cancer. Thus, this research aimed to explore the effect of HCK expression in the survival outcomes of breast cancer patients. Further, we explored the molecular functions and regulation pathways of HCK based on bioinformation tools.

## Methods

### Patients and specimens

The research included patients diagnosed as invasive ductal carcinoma from May 2006 to April 2008 at China Medical University. All specimens were histologically confirmed to be invasive ductal carcinoma, and the patients received surgery and standard treatment. The inclusion criteria were: (1) complete clinicopathological information; (2) no metastasis at the time of operation; and (3) more than 10 axillary lymph nodes were dissected and pathologically evaluated. Patients without complete clinicopathological data, those who did not received standard adjuvant treatment, and with unknown survival status were excluded. The serum, fresh breast cancer tissues, non-cancer tissues, primary and metastatic breast cancer specimens were also collected from China Medical University. No patient received adjuvant chemotherapy or radiotherapy before the surgery, and all patients had 5 years' follow-up at least. Disease-free survival (DFS) was defined from the time of the surgery to the time of local recurrence/distant organ metastasis happened. Overall survival (OS) was defined from the performance of surgery to the time of death. The survival status was determined via outpatient physical examination and interviews or telephone calls. This protocol was approved by the Institutional Review Board of China Medical University. The institution review board (IRB) number is 2018PS336K.

### Pathologic evaluation

Tumor resection and evaluation, including histological grade, estrogen receptor (ER) status, progesterone receptor (PR) status, human epidermal growth factor receptor 2 (HER2) status, and the Ki67 index, were conducted according to the guidelines of China. The molecular types of breast cancer, Luminal A type, Luminal B type, HER2-positive type, and triple-negative type; were defined according to NCCN Guidelines 21. The cut-off values for ER and PR expression was defined as 10% 22. HER2 positive was defined as a score of 3+ by immunohistochemistry (IHC) or score of 2+ by fluorescence *in situ* hybridization 23. Lastly, the cut-off value for the Ki67 index was 20% 24.

### Immunohistochemistry analysis

Breast cancer specimens, fresh breast cancer tissues, fresh non-cancer specimens, primary and metastatic breast cancer specimens were fixed in 4% formaldehyde. Then they were embedded in paraffin. After that they were sliced to 5-μm section. These sections were deparaffinized by xylene and then rehydrated by a graded ethanol series followed by Tris-buffered saline (TBS). After that they were incubated with a primary antibody against HCK (1:150; novus, NBP1-47514) at 4°C overnight. On the second day, they were washed with TBS three times and incubated with a secondary antibody (Gene Tech Co. Ltd., Shanghai, China) at 37°C for approximately 45-60 min and incubated again with a type of DAB kit (Gene Tech Co. Ltd.) for 5-10 min.

HCK expression was semi-quantitatively scored according to the following parameters: 0, if <1% of breast cancer cells expressed cytoplasmic or membrane HCK; 1+, if HCK was expressed in ≥1% but <5% of breast cancer cells; 2+, if HCK was expressed in ≥5% but <10% of breast cancer cells; and 3+, if HCK was expressed in ≥10% of cancer cells. Score 1+, 2+ and 3+ were all considered for HCK expression 25.

### Real-time PCR

Total RNA from the four breast cancer cell lines and 20 breast cancer/cancer-side specimens were isolated using Trizol (Solarbio; R1100), then reversed-transcribed using a cDNA synthesis kit (TaKaRa), following manufacturer protocol. This experiment was performed with the SYBR Green PCR Master Mix and appropriate primers (*HCK*: Forward, 5ʹ-CAGCCGGAAGGACGCAGAGC-3ʹ and Reverse, 5ʹ-AGCCCCCGTTGTCCAGGGTC-3ʹ; β-actin: Forward, 5ʹ-GGCTGTATTCCCCTCCATCG-3ʹ and Reverse, 5ʹ-CCAGTTGGTAACAATGCCATGT-3ʹ) on the Fast-Real-Time PCR System. The thermocycling schedule was 95°C for 30 s, followed by 40 cycles of 95°C for 3 s, and 60°C for 30 s. The level of relative mRNA was calculated by using the 2-ΔΔCt method 26.

### Western blotting

Breast cancer cells, fresh breast cancer tissues, and cancer-side tissues were harvested via trypsinization and then lysed in NP40 lysis buffer. The cell and tissue lysates were centrifuged at high speed to pellet any insoluble materials. The individual cell or tissue lysate (45 μg/lane) was separated via sodium dodecyl sulfate polyacrylamide gel electrophoresis on 12% gels and proteins were transferred to PVDF membranes (0.45 um). Then, after blocked by 5% fat-free dry milk in TBS solution that contained 0.1% Tween 20 for approximately 2 hours, these membranes were incubated with a kind of mouse monoclonal anti-human HCK antibody (1:1000, novus, NBP1-47515ss) or an anti-beta tubulin antibody (1:1000, Proteintech company) over gentle shaking overnight at 4°C. On the second day, after washed with TBS three times, these membranes were incubated with a goat anti-mouse IgG (Zhong Shan Jin Qiao Co. Ltd.) and goat anti-rabbit IgG (Zhong Shan Jin Qiao Co. Ltd.) for one hour and a half at 25°C, and immunoreactive bands were visualized using an enhanced chemiluminescent reagent.

### Elisa

HCK level in the serum of 40 breast cancer patients and 40 benign breast disease patients were quantified via the HCK Elisa kit (MM-50766H2, MEIMIAN, Co, Ltd.) in accordance with the manufacturer's protocol.

### Breast cancer cell and cell culture

Human breast cancer cell lines BT549, MDA-MB-231, MCF7, and SKBR3 were purchased from ATCC. BT549 were cultured in 1640 medium (Biological Industries, Cromwell, USA). MDA-MB-231 were cultured in Leibovitz's L15 medium (Thermo Fisher Scientific, Carlsbad, USA). MCF7 were cultured in DMEM medium (Biological Industries, Cromwell, USA). SKBR3 were cultured in 5A medium (Biological Industries, Cromwell, USA). All these cells were incubated at an atmosphere of 37°C and 5% CO_2_.

### Bioinformatic database mining

The Oncomine database (www.oncomine.org), the Cancer Cell Line Encyclopedia (CCLE) database (www.portals.broadinstitute.org/ccle) and the Gene expression-based Outcome for Breast cancer Online (GOBO) database (http://co.bmc.lu.se/gobo/gsa.pl), were mined to analyze the level of *HCK* mRNA transcripts in breast cancer.

The GEPIA dataset analysis (http://gepia.cancer-pku.cn/) was applied to analysis the level of *HCK* mRNA expression in tumor tissues and cancer-side tissues of breast cancer 27. The UALCAN database (ualcan.path.uab.edu/index.html) was applied to analysis the relationships between the level of *HCK* mRNA expression and some clinicopathological characteristics. Furthermore, the level of *HCK* promoter methylation and some clinicopathological characteristics was also analyzed by UALCAN 28. The cBioPortal database (http://www.cbioportal.org) was used to analysis the *HCK* genetic variations 29.

Genes co-expressed with *HCK* were screened from the Coexpedia website (http://www.coexpedia.org/) 30. Potential biological pathways and processes were further predicted in FunRich 2.1.2 software to explore HCK molecular mechanisms.

For an in-depth exploration of the relationship, we further used the STRING database version 10.0 31. STRING is a database of known and predicted protein-protein associations that have been established based on several information sources, including curated databases, experimental/biochemical data, PubMed abstracts, and others 32. Using the HCK as input parameter, we used STRING to search proteins that interact with HCK. The default scoring threshold of interaction was 0.4, and the subnetwork constructed with those genes which were interacted with *HCK* was further extracted. And the *HCK* driving genes and genes that interacted with *HCK* were constructed into a network. Then, we used the functions of STRING database to conduct gene ontology (GO) enrichment and Kyoto Encyclopedia of Genes and Genomes (KEGG) pathway analyses of all selected genes.

TISIDB database (http://cis.hku.hk/TISIDB) was also used to explore the relationships between HCK and lymphocytes, immunomodulator and chemokine 33. The expression of HCK in different immune subgroup and molecular subgroup of breast cancer was also explored.

### Statistical analysis

Differences in age, other disease, histological grade, menopausal status, ER expression status, PR expression status, HER2 expression status, Ki67 index, distant metastasis, and death among the breast cancer patients were analyzed by Chi-square test. DFS, OS, tumor size, and number of positive axillary lymph nodes (PALNs) were determined via independent-sample *t*-tests.

Student's *t*-test was used to compare across cancer specimens from the two databases and normal control datasets. Survival curves were performed by using the Kaplan-Meier test via SPSS 19.0 software. Univariate and multivariate Cox regressions were used to find independent predictors of prognosis associated with DFS and OS. In addition, hazard ratios (HRs) and 95% confidence intervals (CIs) were also calculated. All these* P* values were two-sided, and significance was at *P*<0.05, except for analyses pertaining to database mining, which was significant at *P*<0.01 (two-fold change or higher). Unless otherwise stated, analyses were performed by SPSS 19.0.

## Results

### Analysis of differential expression of *HCK* in breast cancer based on some bioinformatic databases

**Figure 1:**
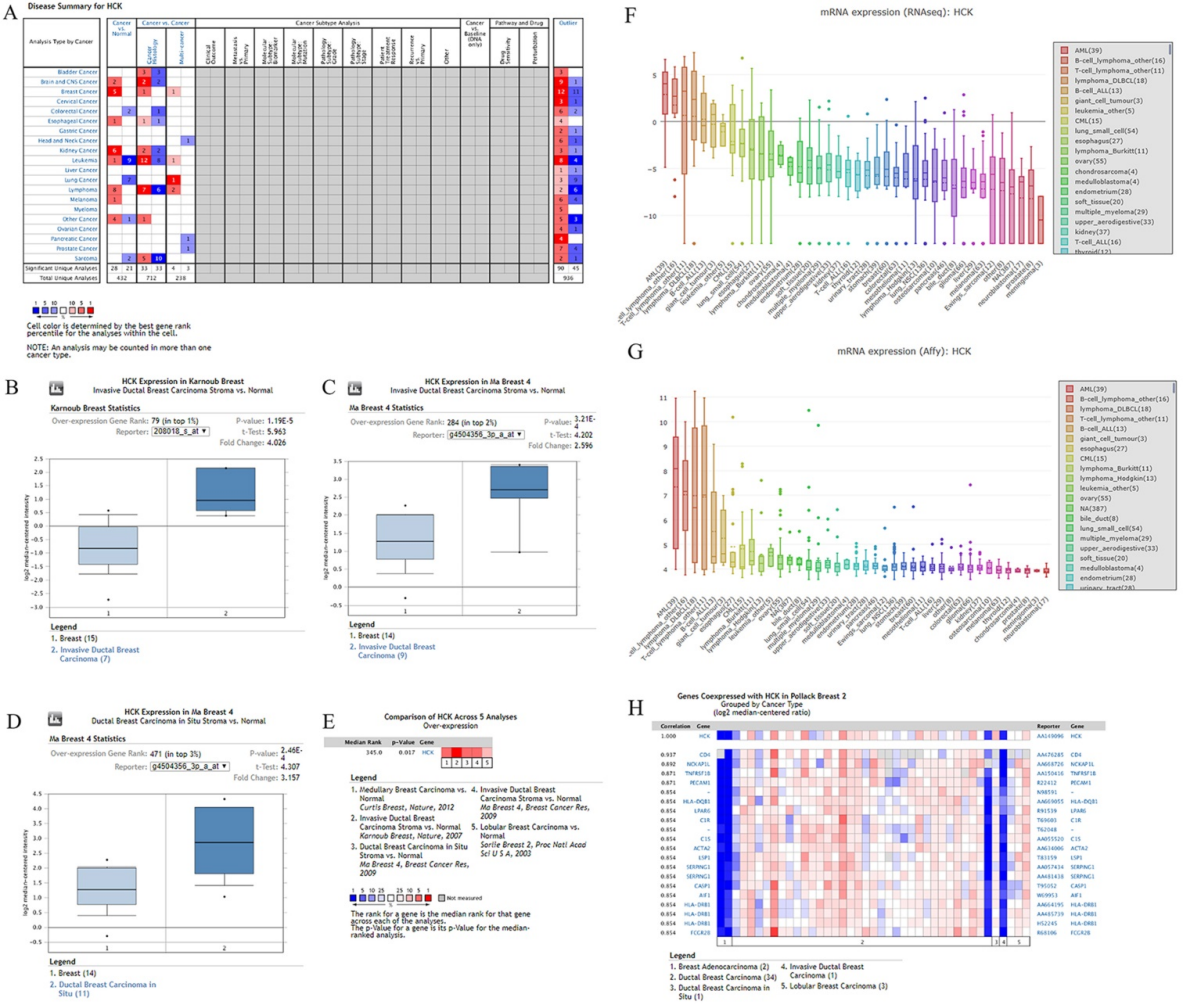
** Oncomine and CCLE analysis of *HCK* expression in breast cancer. A:** The level of *HCK* mRNA transcripts in different tumor types. The graph shows the number of datasets with statistically significant mRNA target genes (cancer to non-cancer tissue, cancer to cancer) with increased (red) or reduced expression (blue). The *P* value threshold is 0.01. The numbers in each cell represent the number of analyses that meet the threshold in these analyses and the cancer types. **B-D:** Comparison of the level of *HCK* mRNA transcripts between breast cancer tissues and normal breast tissues in *Karonub Breast* and* Ma Breast 4* group. **E:** Meta-analysis of multiple datasets for a more comprehensive comparison of *HCK* mRNA level between breast cancer tissues and normal breast tissues. **F-G:** The comparison of the level of *HCK* mRNA transcripts in breast cell line with other different cell lines from the CCLE analysis. **H:** The gene co-expression analysis of *HCK* in breast cancer.

Differential *HCK* expression was found in 20 human cancers based on the database, including solid tumors (Fig. 1A). Oncomine analysis revealed that the level of *HCK* mRNA transcripts was significantly higher in breast cancer than in non-cancer samples. We then further evaluated the level of *HCK* mRNA transcripts in some single studies. In the study by Karnoub breast study, *HCK* transcripts were increased by 4.026-fold in invasive ductal breast carcinoma samples compared with normal tissues (*P*=1.19 E-5) (Fig. 1B). Meanwhile, in another dataset from the study by Ma Breast study, we found a 2.596-fold increase in the level of *HCK* mRNA transcripts in invasive ductal breast carcinoma stroma (*P*=3.21 E-4) (Fig. 1C) and 3.157-fold increase in *in situ* samples of ductal breast carcinoma compared with non-cancer tissues (*P*=2.46 E-6) (Fig. 1D). To obtain a more comprehensive conclusion, we conducted a meta-analysis of multiple datasets, and the results showed a significantly increased *HCK* mRNA expression in breast cancer tissues compared with normal tissues (Fig. 1E). Meanwhile, results of the CCLE analysis showed the level of *HCK* mRNA transcripts in breast cell line ranks 24^th^ and 26^th^ among many cell lines (Fig. 1F, 1G). Consequently, we performed the co-expression analysis of *HCK* using the Oncomine database (Fig. 1H). In this analysis, HCK expression was found to be most significantly correlated with CD4 (r=0.937). These results suggested that HCK may act with CD4 to play a potential important role in regulating the biological behaviors of breast cancer.

### Relationships between HCK expression and clinicopathological characteristics

**Figure 2:**
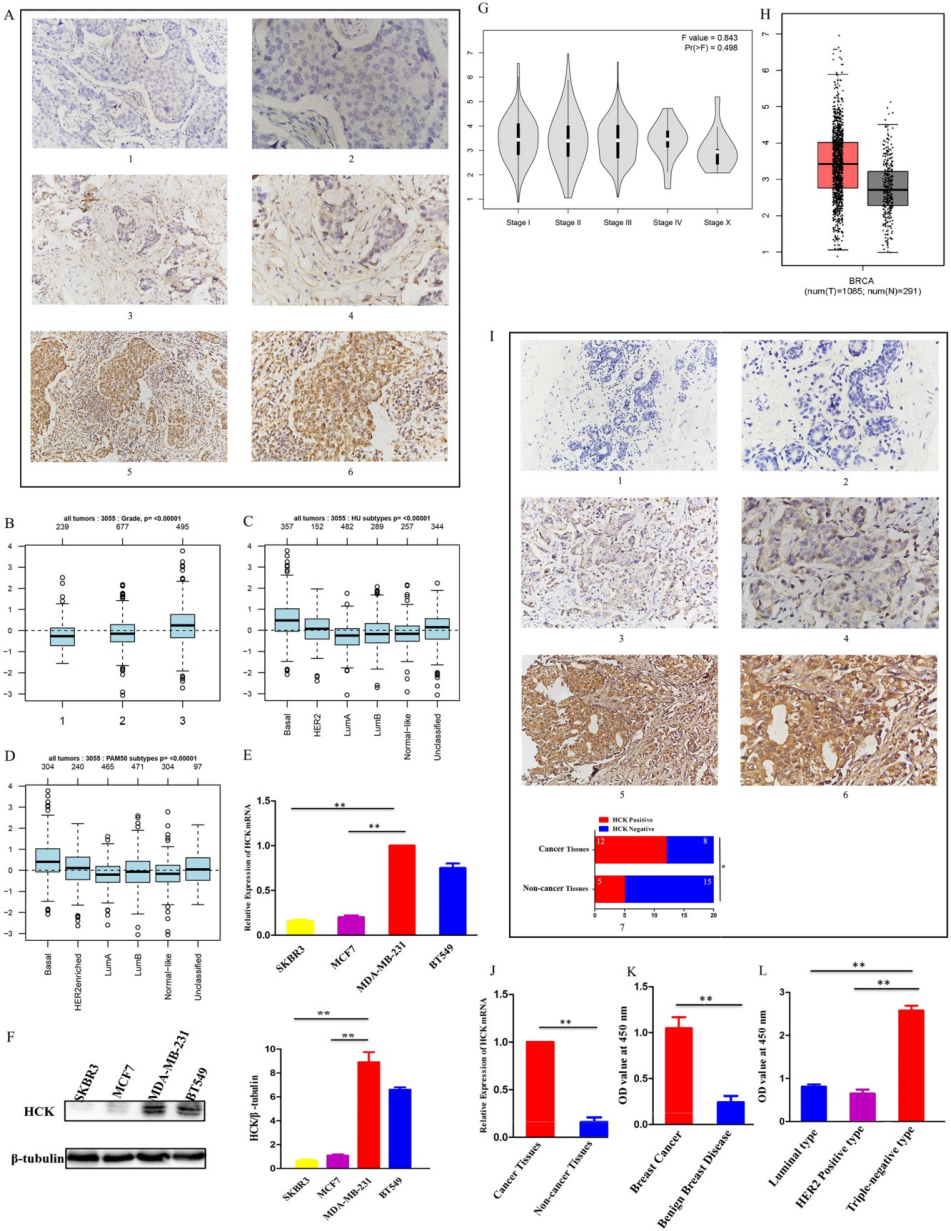
** Relationships between HCK expression and clinicopathological characteristics. A:** (1) and (2): Representative negative HCK immunohistochemical staining results in the breast cancer specimens. (1): ×200 magnification; (2): ×400 magnification. (3) and (4): Representative weak positive HCK immunohistochemical staining in the breast cancer specimens. (3): ×200 magnification; (4): ×400 magnification. (5) and (6): Representative strong positive HCK immunohistochemical staining in the breast cancer specimens. **B:** The level of *HCK* mRNA transcripts in breast cancer patients with different tumor grades in GOBO database. **C-D:** The level of *HCK* mRNA transcripts in breast cancer patients with different molecular types in GOBO database. **E:** The relative *HCK* mRNA transcripts level in BT549, MDA-MB-231, MCF7, and SKBR3 cell lines determined via real-time PCR and quantitatively analyzed. **F:** The relative HCK expression in BT549, MDA-MB-231, MCF7, and SKBR3 cell lines determined via western blot and quantitatively analyzed. **G:** The level of *HCK* mRNA transcripts in breast cancer patients with different tumor stage in GEPIA database. **H:** The level of *HCK* mRNA transcripts in cancer tissues and normal tissues in GEPIA database. **I:** (1) and (2): Representative negative HCK immunohistochemical staining in fresh breast cancer tissues. (1): ×200 magnification; (2): ×400 magnification. (3) and (4): Representative weak positive HCK immunohistochemical staining in the breast cancer specimens. (3): ×200 magnification; (4): ×400 magnification. (5) and (6): Representative strong positive HCK immunohistochemical staining results in fresh cancer-side tissues. (5): ×200 magnification; (6): ×400 magnification. (7): The rate of HCK expression in breast cancer tissues was significantly higher compared with non-cancer tissues (*P*=0.025). **J:** The relative *HCK* mRNA transcripts level in cancer tissues and non-cancer tissues determined via real-time PCR and quantitatively analyzed. **K:** The HCK level in serum of patients with breast cancer and patients with benign breast disease determined via Elisa and quantitatively analyzed. **L:** The HCK level in serum of different molecular subtype breast cancer patients determined via Elisa and quantitatively analyzed.

Analysis of the 87 breast cancer patients showed that HCK expression was related to a larger tumor size (*P*=0.008) and a greater number of PALNs (*P*=0.006), but not with other clinicopathological characteristics (Table 1). Representative images of HCK expression in these breast cancer specimens were shown in Figure 2A. Results of the GOBO database analysis also showed that the level of *HCK* mRNA transcripts was higher in tumors with grade 3 than in tumors with grade 1 and grade 2 (Fig. 2B). The database also showed that the level of *HCK* mRNA transcripts was higher in basal type breast cancer than that in HER2+ or luminal A/B type breast cancer (Fig. 2C, 2D). This result was consistent to our *in vitro* experiment of cell lines. We found that the level of *HCK* mRNA transcripts and HCK protein expression were both higher in highly invasive and metastatic BT549 and MDA-MB-231 triple-negative breast cancer cells than that in less invasive and metastatic SKBR3 and MCF7 breast cancer cells (Fig. 2E, 2F). By using CEPIA dataset, we did not find a significantly difference of *HCK* mRNA expression in patients with different stage (Fig. 2G). However, we found that the level of *HCK* mRNA expression was significantly higher in cancer tissues than that in non-cancer normal tissues (*P*<0.01, Fig. 2H). IHC of HCK expression in 20 breast cancer tissues and 20 breast non-cancer tissues collected in our hospital also showed that HCK expression was significantly higher in cancer tissues than that in non-cancer tissues (*P*=0.025, Fig. 2I). The level of *HCK* mRNA transcripts was also significantly higher in cancer tissues than that in non-cancer tissues in our specimens (*P*<0.01, Fig. 2J). We further evaluated the level of HCK in serum of 40 breast cancer patients and 40 patients with benign breast disease by Elisa test. The level of HCK in serum was significantly higher in patients with breast cancer compared with patients with benign breast disease (*P*<0.01, Fig. 2K). Among these 40 breast cancer patients, the level of HCK was higher in 6 triple-negative breast cancer patients than in 27 luminal type and 7 HER2 positive breast cancer patients (Fig. 2L).

**Figure 3:**
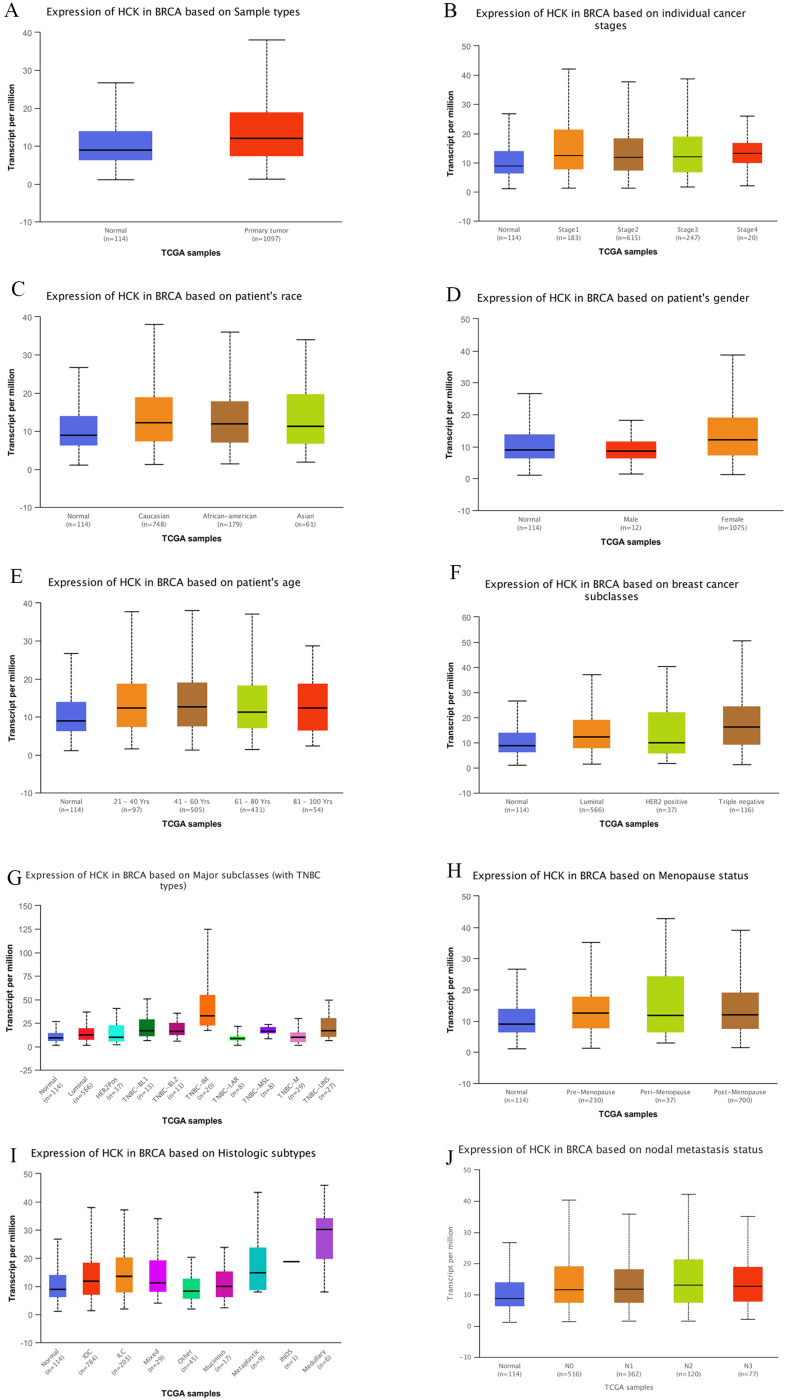
** UALCAN analysis of *HCK* mRNA expression in breast cancer.** Expression of *HCK* in breast cancer based on different sample types (**A**), individual cancer stages (**B**), patient's race (**C**), patient's gender (**D**), patient's age (**E**), breast cancer subclasses (**F**), Major subclasses (with TNBC types) (**G**), Menopause status (**H**), Histologic subtypes (**I**), and nodal metastasis status (**J**).

In addition, we also explored the level of *HCK* mRNA expression in breast cancer using UALCAN dataset. Consistent to above results, the level of *HCK* mRNA expression was significantly higher than in non-cancer tissues (*P*<0.01, Fig. 3A). In different stage of breast cancer, patients with stage 4 had highest *HCK* mRNA expression and patients with stage 2 had lowest *HCK* mRNA expression. But the difference was not significant (Fig. 3B). In different race of breast cancer patients, Caucasian patients had highest *HCK* mRNA expression, Asian patients had lowest *HCK* mRNA expression; the difference was also not significant (Fig. 3C). In different gender of breast cancer patients, female patients had higher *HCK* mRNA expression than male patients (*P*<0.01, Fig. 3D). In different patients' age groups, patients aged 41-60 years old had highest *HCK* mRNA expression, whilst patients in 61-80 years old had lowest *HCK* mRNA expression. But the difference was not significant (Fig. 3E). In different molecular type of breast cancer, triple negative patients had higher *HCK* mRNA expression than HER2 positive and luminal type patients (*P*<0.01, Fig. 3F). We further found that patients with triple negative breast cancer-immunomodulatory subclasses had the highest *HCK* mRNA expression among all these subclasses (*P*<0.01, Fig. 3G). In patients with different menopause status, pre-menopause patients had highest *HCK* mRNA expression than other status, but the difference was not significant (Fig. 3H). In patients with different histologic subtypes, medullary type had highest *HCK* mRNA expression than others (*P*<0.01, Fig. 3I). At last, in patients with different lymph node status, N2 patients had highest *HCK* mRNA expression than others, but the difference was not significant (Fig. 3J).

### Relationships between *HCK* promoter methylation and clinicopathological characteristics

**Figure 4:**
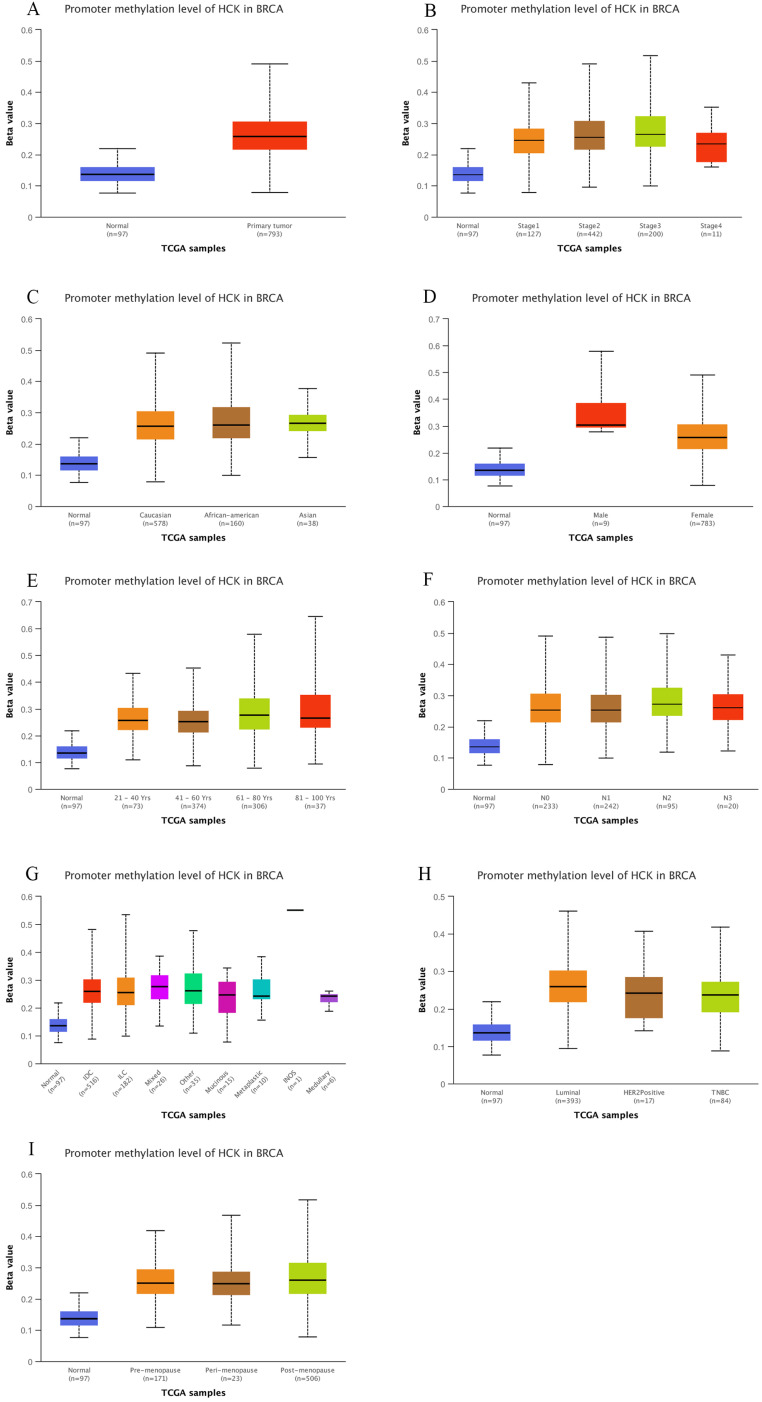
** UALCAN analysis of *HCK* promoter methylation in breast cancer.** The level of *HCK* promoter methylation in breast cancer was compared based on different sample types (A), individual cancer stages (B), patient's race (C), patient's gender (D), patient's age (E), nodal metastasis status (F), Histologic subtypes (G), breast cancer subclasses (H), and Menopause status (I). **A:** The level of *HCK* promoter methylation was significantly higher in primary tumor than in non-cancer tissues (*P*<0.01). In breast cancer patients, **B:** Patients with stage 3 had highest level of *HCK* promoter methylation and patients with stage 4 had lowest level of *HCK* promoter methylation, but the difference was not significant. **C:** There was also no significant difference of *HCK* promoter methylation in patients with different race. **D:** Male patients had higher level of *HCK* promoter methylation than female patients (*P*<0.01). **E:** Patients 61-80 years old had highest level of *HCK* promoter methylation, patients in 41-60 years old had lowest level of *HCK* promoter methylation (*P*<0.01). **F:** N2 patients had highest level of *HCK* promoter methylation, N1 patients had the lowest level of *HCK* promoter methylation. But the difference was not significant. **G:** Mixed type had the highest level of *HCK* promoter methylation than others, but the difference was not significant. **H:** Luminal type patients had the highest level of *HCK* promoter methylation, triple negative type patients had the lowest level of *HCK* promoter methylation (*P*<0.01). **I:** Post-menopause patients had the highest level of *HCK* promoter methylation, pre-menopause patients had the lowest level of *HCK* promoter methylation (*P*<0.01).

UALCAN dataset also gave us the chance to explore if promoter methylation of *HCK* was related to the clinicopathological characteristics of breast cancer patients, so as to promote the development of breast cancer. Using the dataset, we found that the level of *HCK* promoter methylation was significantly higher in primary tumor than in non-cancer tissues (*P*<0.01, Fig. 4A). In patients with different stage, patients with stage 3 had highest level of *HCK* promoter methylation and patients with stage 4 had lowest level of *HCK* promoter methylation, but the difference was not significant (Fig. 4B). There was also no significant difference of *HCK* promoter methylation in patients with different race (Fig. 4C). In different gender of breast cancer patients, male patients had higher level of *HCK* promoter methylation than female patients (*P*<0.01, Fig. 4D). In different patients' age, patients in 61-80 years old had highest level of *HCK* promoter methylation, patients in 41-60 years old had lowest level of *HCK* promoter methylation (*P*<0.01, Fig. 4E). In patients with different lymph node status, N2 patients had highest level of *HCK* promoter methylation, N1 patients had the lowest level of *HCK* promoter methylation. But the difference was not significant (Fig. 4F). In patients with different histologic subtypes, mixed type had the highest level of *HCK* promoter methylation than others, but the difference was not significant (Fig. 4G). In patients with different molecular types, Luminal type patients had the highest level of *HCK* promoter methylation, triple negative type patients had the lowest level of *HCK* promoter methylation (*P*<0.01, Fig, 4H). At last, in patients with different menopause status, post-menopause patients had the highest level of *HCK* promoter methylation, pre-menopause patients had the lowest level of *HCK* promoter methylation (*P*<0.01, Fig. 4I). All the findings proved that *HCK* promoter methylation may contribute to the development of breast cancer.

### Mutation, amplification and fusion of *HCK* gene in breast cancer

**Figure 5:**
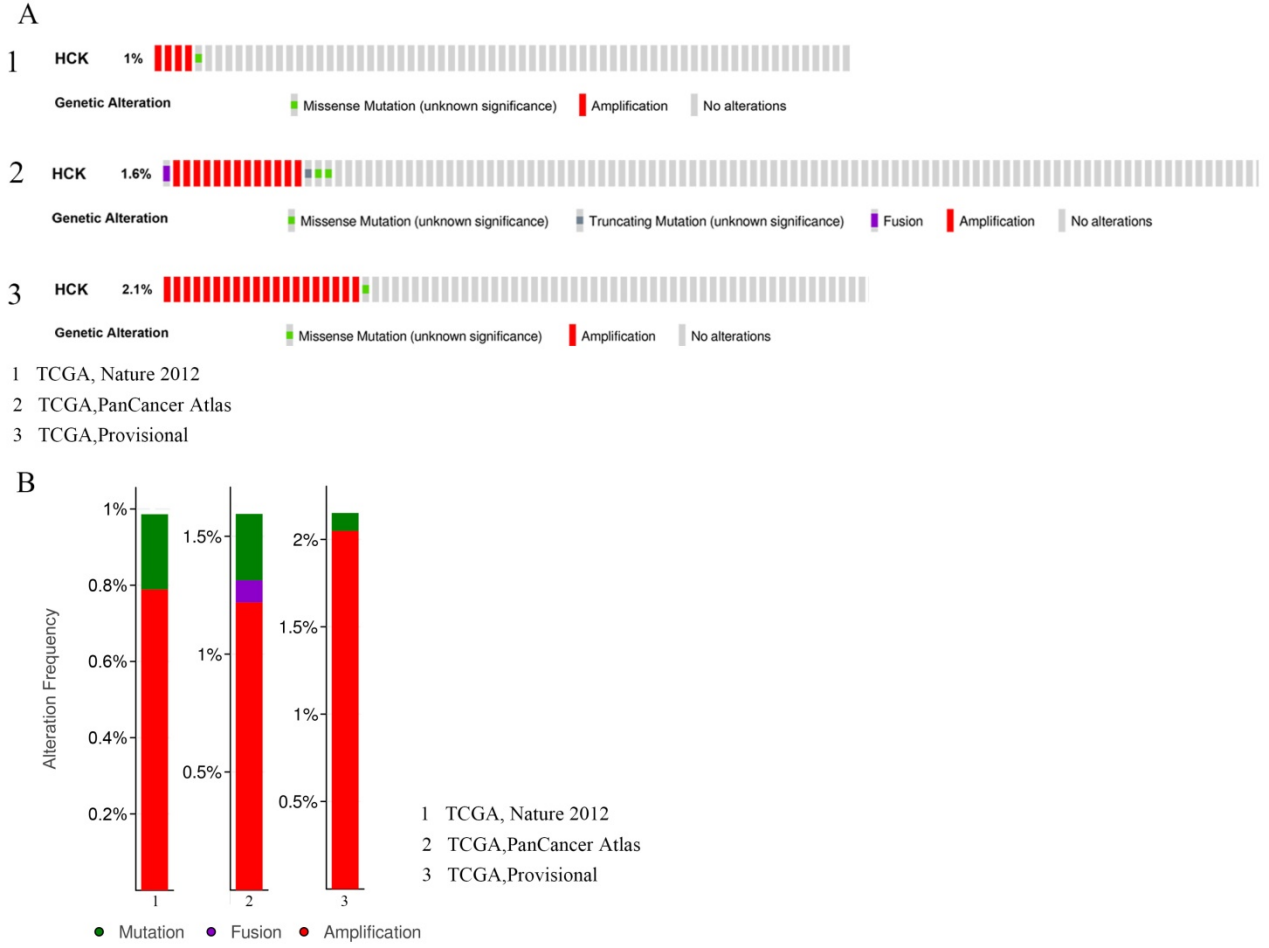
** Analyses of genetic variations of *HCK* gene in breast cancer by cBioPortal database. A:** OncoPrint visual summary of genetic variations of *HCK* gene. **B:** Analyses of genetic variations of *HCK* reported in different studies.

Genetic variations of *HCK* in 2549 cases which were retrieved from 3 studies (507 cases from TCGA, Nature 2012; 1066 cases from TCGA, PanCancer Atlas; and 976 cases from TCGA, Provisional) were analyzed by applying the cBioPortal database (Fig. 5). In these 507 cases from TCGA, Nature 2012, the incidence ratio of *HCK* mutation was 0.2% and amplification was 0.79%. In these 1066 cases from TCGA, PanCancer Atlas, the incidence ratio of *HCK* mutation, fusion and amplification were 0.28%, 0.09% and 1.22%, respectively. At last, in these 976 cases from TCGA, Provisional, the incidence ratio of *HCK* mutation was 0.1% and amplification was 2.05%.

### HCK expression in the prognosis of breast cancer patients

**Figure 6:**
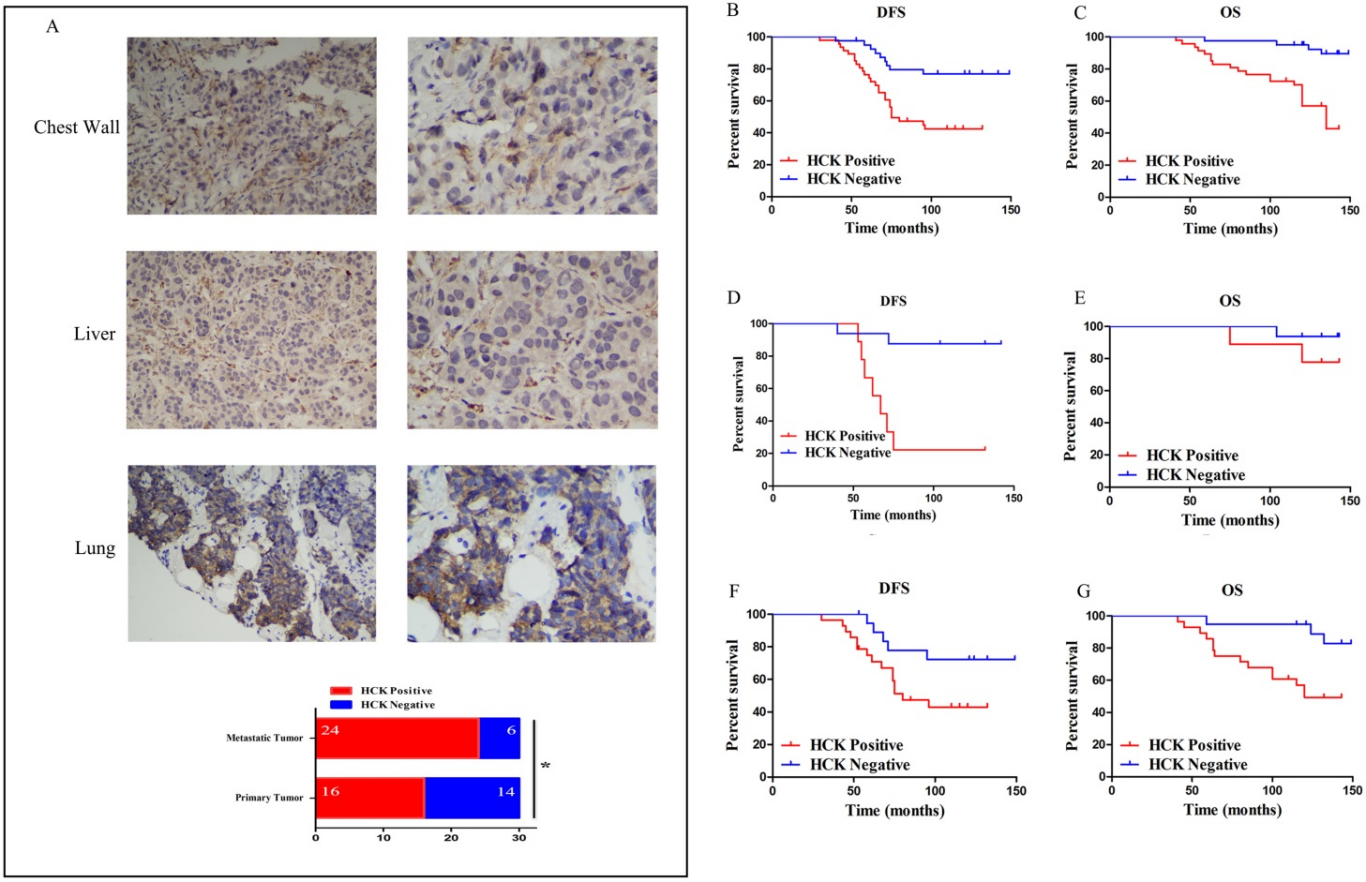
** Effect of HCK expression in the survival outcomes of breast cancer patients. A:** (1) and (2): Representative positive HCK immunohistochemical staining of metastatic tumor in chest wall. (1): ×200 magnification; (2): ×400 magnification. (3) and (4): Representative positive HCK immunohistochemical staining of metastatic tumor in liver. (3): ×200 magnification; (4): ×400 magnification. (5) and (6): Representative positive HCK immunohistochemical staining results of metastatic tumor in lung. (5): ×200 magnification; (6): ×400 magnification. (7): The rate of HCK expression in metastatic tumor was significantly higher than primary tumor (*P*=0.028). **B:** DFS survival curve based on HCK expression in the overall population. **C:** OS survival curve based on HCK expression in the overall population. **D:** DFS survival curve in luminal A type patients based on HCK expression. **E:** OS survival curve in luminal A type patients based on HCK expression. F: DFS survival curve in luminal B type patients based on HCK expression. G: OS survival curve in luminal B type patients based on HCK expression.

First, to understand the role of HCK expression in the metastasis of breast cancer patients, the expression of HCK was detected by immunohistochemistry in 30 pairs of primary and metastatic breast cancer specimens. The result presented that the ratio of HCK expression was significantly higher in metastatic tumors (like in chest wall, liver and lung) than that in primary breast cancer (*P*=0.028; Fig.6A). This finding indicated that HCK may promote metastasis of breast cancer.

Next, our analysis of HCK expression in the prognosis of breast cancer showed that it was related to distant metastasis and death (*P*=0.002; *P*<0.001). The ratio of distant organ metastasis and death were higher in patients with HCK expression than in those without expression (55.3% and 44.7% vs 22.5% and 10.0%). Patients with HCK expression also had a significantly shorter DFS and OS (*P*<0.001; *P*=0.001). The average DFS and OS was 88.34 months and 112.17 months in patients with HCK expression and 11.538 months and 130.28 months in patients without HCK expression, respectively. These data preliminarily indicate that HCK expression was associated with a negative survival outcome in breast cancer patients (Table 2).

In these 87 breast cancer patients, HCK expression was related to lower DFS and OS on Kaplan-Meier analysis. The difference was also significant (DFS: *P*=0.001, Fig. 6B; OS: *P*<0.001, Fig. 6C).

Then, we analyzed the role of HCK expression in patients with different molecular types. However, because there were only 7 patients with HER2+ breast cancer and 8 patients with triple-negative breast cancer, we did not perform survival analysis in this two groups. The role of HCK expression was only analyzed in luminal A type and luminal B type patients. Luminal A type patients with HCK expression had a significantly shorter DFS than those patients without HCK expression (*P*<0.001, Fig. 6D); however, there was no significant difference in OS (*P*=0.245, Fig. 6E). With respect to luminal B type patients, those with HCK expression had significantly lower DFS (*P*=0.049, Fig. 6F) and OS (*P*=0.012, Fig. 6G). These results predicted that HCK expression was significantly related to a worse prognosis of breast cancer patients.

### Subgroup analysis on the effect of HCK expression on survival outcomes

To thoroughly explore the effect of HCK expression on the survival outcome of breast cancer patients, these patients were divided into the following five subgroups.

**Figure 7:**
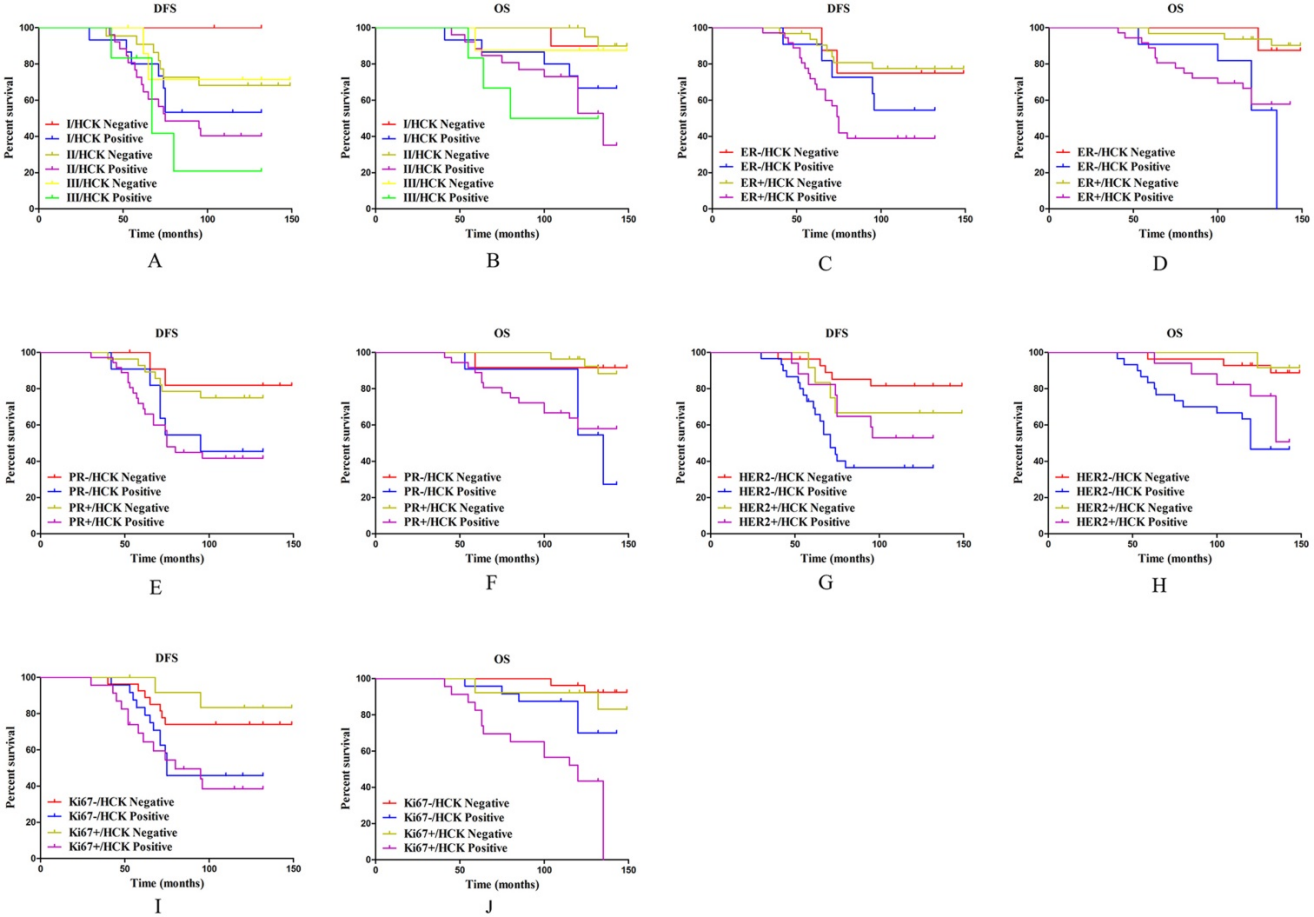
** Subgroup analysis on the effect of HCK expression on survival outcomes. A:** DFS survival curve in different histological grade of breast cancer based on HCK expression. **B:** OS survival curve in different histological grade of breast cancer based on HCK expression. **C:** DFS survival curve in different ER status based on HCK expression. **D:** OS survival curve in different ER status based on HCK expression. **E:** DFS survival curve in different PR status based on HCK expression. **F:** OS survival curve in different PR status based on HCK expression. **G:** DFS survival curve in different HER2 status based on HCK expression. **H:** OS survival curve in different HER2 status based on HCK expression. **I:** DFS survival curve in different Ki67 indices based on HCK expression. **J:** OS survival curve in different Ki67 indices based on HCK expression.

In the histological grade subgroups, as for DFS, except in histological grade 3 patients, patients with HCK expression did not have significant reduced DFS (*P*=0.160, Fig. 7A), HCK expression significantly shortened DFS in patients with histological grade of 1 (*P*=0.015, Fig. 7A) and 2 (*P*=0.047, Fig. 7A). Meanwhile, only patients with histological grade 2 disease with HCK expression had significantly shorter OS (*P*=0.002, Fig. 7B).

In the ER status subgroups, HCK expression can significantly reduce DFS in patients with ER+ status (*P*=0.001, Fig. 7C), but not in ER- patients (*P*=0.397, Fig. 7C). As for OS, patients with HCK expression had significantly lower OS regardless of ER- (*P*=0.048, Fig. 7D) or ER+ status (*P*=0.002, Fig. 7D).

In the PR status subgroups, HCK expression had a similar effect as in ER status. PR+ patients with HCK expression had a significantly shorter DFS and OS than patients without HCK expression (*P*=0.008, Fig. 7E; *P*=0.004, Fig. 7F). Meanwhile, in PR- patients, those with HCK expression had significantly lower OS (*P*=0.024, Fig. 7F), but not DFS (*P*=0.081, Fig. 7E).

In the HER2 status subgroups, HCK expression can also reduce DFS (*P*<0.001, Fig. 7G) and OS in HER2-patients (*P*=0.001, Fig. 7H), but not in HER2+ patients (*P*=0.554, Fig. 4G; *P*=0.142, Fig. 4H).

Finally, in the Ki67 index subgroups, the DFS was significantly shorter in high Ki67 index patients with HCK expression (*P*=0.011, Fig. 7I), but not in those with low Ki67 index (*P*=0.051, Fig. 7I). For OS, patients with HCK expression had a significantly shorter OS regardless of Ki67 index (*P*=0.037, Fig. 7J; *P*=0.012, Fig. 7J).

### Predictive factors associated with DFS and OS

To explore the independent predictors of breast cancer patients' survival outcome, Cox regression analysis were utilized to analyze the clinicopathological characteristics associated with DFS and OS (Tables 3 & 4).

For DFS, tumor size and HCK expression were related to DFS in univariate cox regression analysis (*P*<0.001; *P*=0.003), and thus they were entered into multivariate cox regression analysis. Both tumor size (*P*=0.021) and HCK expression (*P*=0.005) were found to be independent predictors associated with DFS in breast cancer.

For OS, tumor size, Ki67 index, and HCK expression were related to OS (*P*=0.001; *P*=0.004; *P*=0.001) in univariate cox regression analysis, and thus they were entered into multivariate cox regression analysis. These three factors were found to be independent predictors associated with OS (*P*<0.001; *P*=0.006; *P*=0.005). These results clearly support that the expression of HCK is an independent predictor of DFS and OS in breast cancer.

### Exploration of HCK molecular functions and regulation pathways based on bioinformation tools

**Figure 8:**
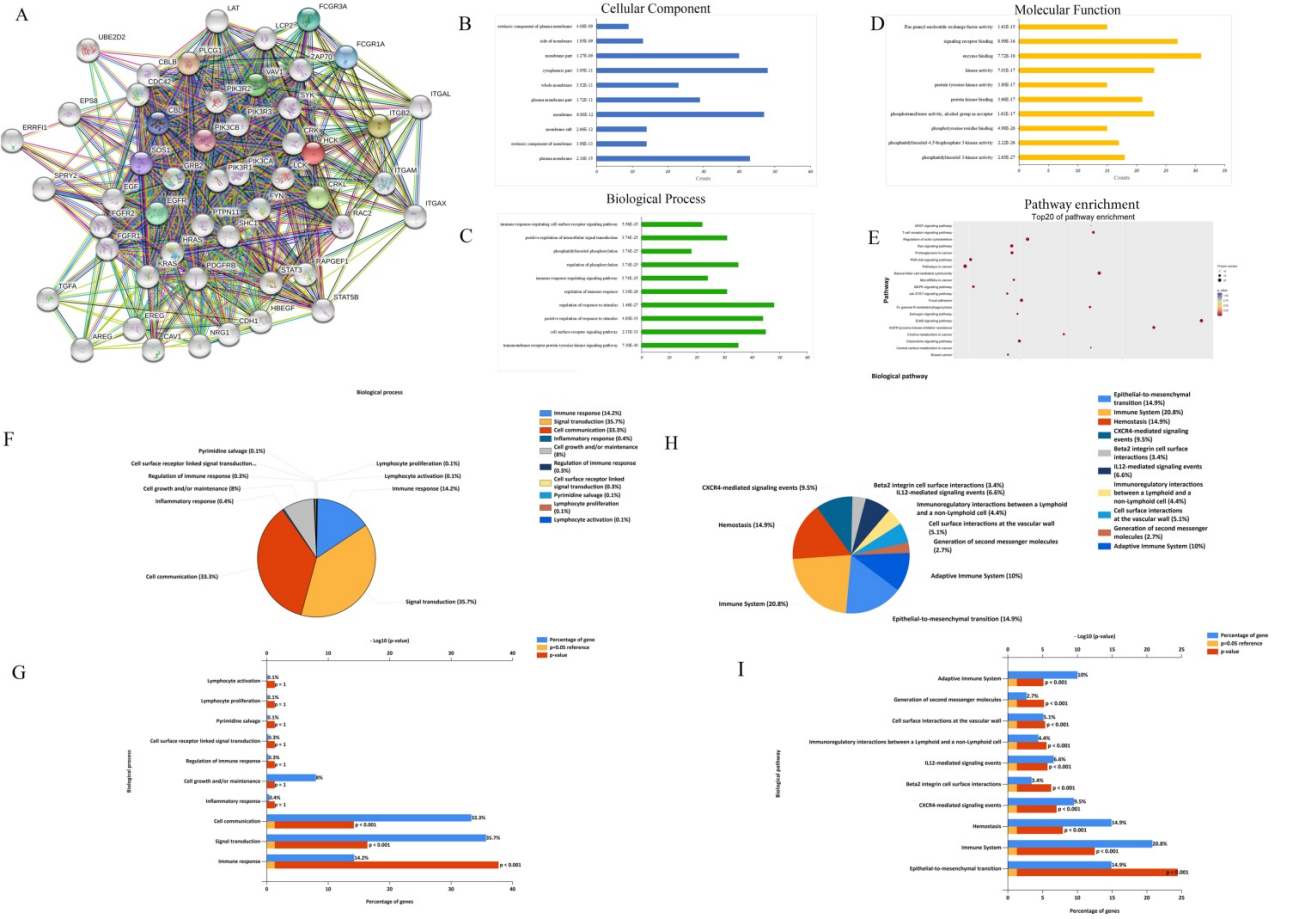
** Exploration of HCK molecular functions and regulation pathways based on bioinformation tools. A:** The STRING interaction network of HCK based on the STRING database. **B:** Cellular component. **C:** GO biological process. **D:** Molecular function analysis. **E:** Pathway enrichment based on KEGG. **F-G:** Potential biological processes identified via FunRich. **H-I:** Potential biological pathways identified via FunRich.

Using the bioinformation databases, we preliminarily explored the HCK molecular function and regulation pathway to provide research directions to explore the mechanism by which HCK regulates the biological behaviors of breast cancer. First, we explored the STRING database to search for genes that interact with HCK (Fig. 8A). These selected genes were then subjected to GO analysis to identify the cellular component (CC) (Fig. 8B), biological process (BP) (Fig. 8C) and molecular function (MF) (Fig. 8D) in which HCK and its interacted genes were involved. The CC analysis presented that these differentially expressed proteins were in extrinsic component of membrane and cytoplasmic part. The BP analysis suggested that these differentially expressed proteins were mainly involved in immune response-regulating signaling pathway, positive regulation of intracellular signal transduction, phosphatidylinositol phosphorylation, and other related process. The MF analysis revealed that these differentially expressed proteins functioned mainly for Ras guanyl-nucleotide exchange factor activity, signaling receptor binding, and other related function. We further performed KEGG pathway analysis to identify the molecular pathway in which HCK and its interacted genes were involved. We present the top 20 pathway enrichments, such as vascular endothelial growth factor signaling pathway and T cell receptor signaling pathway, in Figure 8E.

Further, we extracted several neighboring genes that were related to HCK from Coexpedia to determine the potential molecular regulation mechanisms of HCK in cancer and other diseases (Supp Fig. 1). In this result of supplementary figure 1A-B, we found that HCK may be correlated with CD4 again, which was consist with the result of Figure 1H. The biological processes and biological pathways of *HCK* and related genes identified from Coexpedia were also investigated using FunRich 2.1.2 software. The analysis provided us some novel findings that were not found in the STRING database. The results demonstrated that HCK was involved in the adaptive immune system, generation of second messenger molecules, and other biological pathways. Furthermore, we also found that HCK was involved in lymphocyte activation and proliferation and other biological process. These results are shown in Figure 8F-8I. We believe that these results may help us determine the exact regulatory mechanisms of HCK in cancer and other diseases.

### Regulation of immune-related molecules by HCK in breast cancer

**Figure 9:**
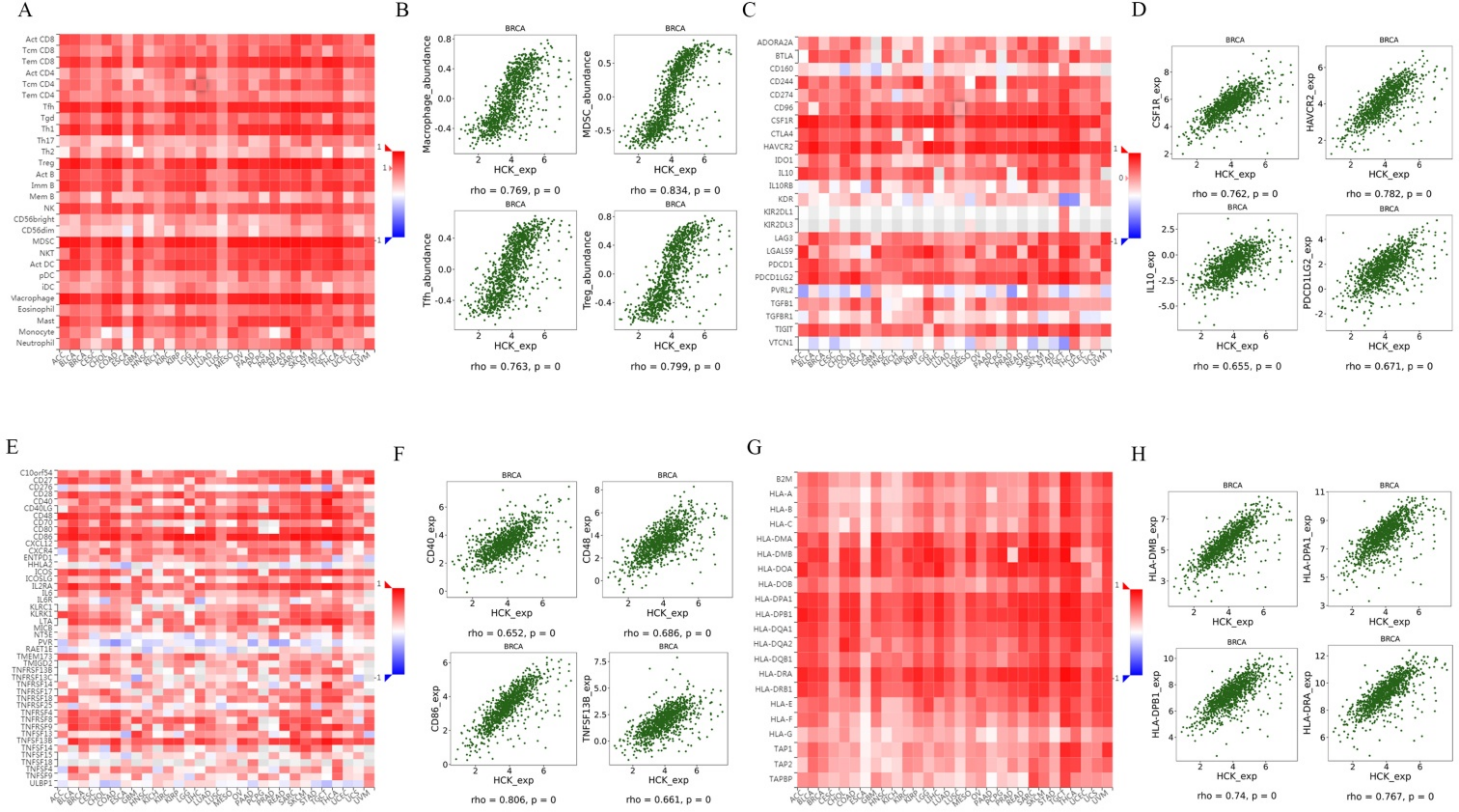
** Regulation of TILs, immunoinhibitors, immunostimulators and MHC molecules by HCK in breast cancer. A:** Relationships between HCK expression and TILs. **B:** Top 4 TILs showing the greatest correlations with HCK expression. **C:** Relationships between HCK expression and immunoinhibitors. **D:** Top 4 immunoinhibitors showing the greatest correlations with HCK expression. **E:** Relationships between HCK expression and immunostimulators. **F:** Top 4 immunostimulators showing the greatest correlations with HCK expression. **G:** Relationships between HCK expression and MHC molecules and. **H:** Top 4 MHC molecules showing the greatest correlations with HCK expression.

STRING database and Coexpedia database both predicted that HCK may be involved in immune response-regulating signaling pathway, T cell receptor signaling pathway, adaptive immune system, lymphocyte activation and proliferation, and other immune-related biological pathways and processes. Therefore, we further explored the regulation of immune-related molecules by HCK in breast cancer using TISIDB database 32. This database can be utilized to analysis the relationships between selected genes and lymphocytes, immunomodulators, and chemokines. Figure 9A showed the relationships between HCK expression and tumor infiltrating lymphocytes (TILs). The top four lymphocytes which were most significantly associated with HCK expression were Macrophage, MDSCs, Tfh, and Treg (Fig. 9B). As for immunomodulators, they can be classified into immunoinhibitors, immunostimulators, and major histocompatibility complex (MHC) molecules. In Figure 9C, we presented the relationships between HCK expression and immunoinhibitors. The top four immunoinhibitors which were most significantly related to HCK expression was CSF1R, HAVCR2, IL10, and PDCD1LG2 (Fig. 9D). Figure 9E presented the correlations between immunostimulators and HCK expression. The immunostimulators which showed the greatest correlations were CD40, CD48, CD86, and TNFSF13B (Fig. 9F). Figure 9G showed the relationships between HCK expression and MHC molecules. The top four MHC molecules that showed the greatest relationships were HLA-MDB, HLA-DPA1, HLA-DPB1, and HLA-DRA (Fig. 9H).

**Figure 10:**
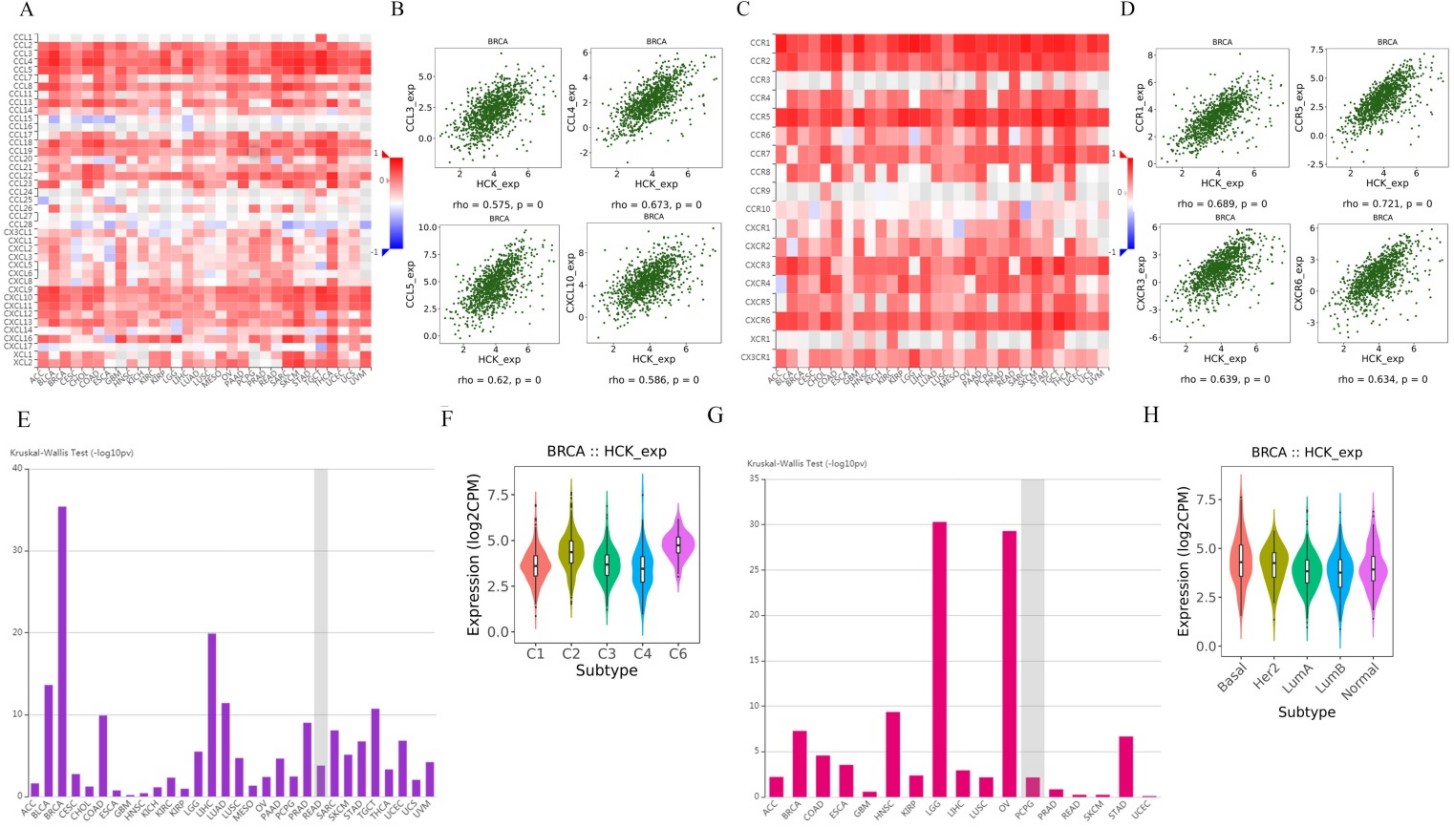
** Regulation of chemokine and receptor by HCK in breast cancer and the expression of HCK in different breast cancer subtype. A:** Relationships between chemokine and HCK expression. **B:** Top 4 chemokines showing the greatest correlations with HCK expression. **C:** Relationships between receptor and HCK expression. **D:** Top 4 receptors showing the greatest correlations with HCK expression. **E-F:** HCK expression in different immune subtype of breast cancer. **G-H:** HCK expression in different molecular subtype of breast cancer.

We further explored the relationships between HCK expression and chemokine and receptor. Figure 10A showed the relationships between HCK expression and chemokine. The chemokines that showed the greatest relationships included CCL3, CCL4, CCL5, and CXCL10 (Fig. 10B). Figure 10C presented the relationships between HCK expression and receptor. The receptors that showed the greatest correlations included CCR1, CCR5, CXCR3 and CXCR6 (Fig. 10D).

At last, we explored the expression of HCK in different immune subtype and molecular subtype by TISIDB database. The expression of HCK was highest in C6 immune subtype of breast cancer (Fig. 10E, 10F). As for in different molecular subtype, the basal subtype showed the highest HCK expression (Fig. 10G, 10H), and the result was consistent to the findings obtained from other databases.

## Discussion

Deregulated expression of HCK has been found in many solid tumors such as pancreatic, prostate, renal, and breast cancers 34-37. HCK belongs to the SFK family, which has been found to promote metastasis in several cancers, such as in CRC 38. However, the role of HCK expression in the survival outcome of cancer patients, particularly those with breast cancer, remains unclear. In this research, we found that HCK expression negatively influence the prognosis of breast cancer patients, and explored the regulation mechanism of HCK in cancer and even in other related disease.

Specifically, Oncomine analysis, GEPIA analysis and UALCAN analysis showed that a higher level of *HCK* mRNA transcripts in cancer tissues than in non-cancer tissues. UALCAN analysis also showed that the level of *HCK* promoter methylation was higher in cancer tissues than in non-cancer tissues. In addition, the cBioPortal database showed the existence of mutation, amplification and fusion of *HCK* gene in breast cancer. Using clinical breast cancer specimens, we found that HCK expression was related to a larger tumor size, a greater number of PALNs, distant metastasis, and even death. HCK expression can also significantly lower DFS and OS. Additionally, HCK expression was higher in highly invasive and metastasis MDA-MB-231 cell lines than in less invasive and metastasis SKBR3 and MCF7 cell lines, indicating that HCK may be related to invasion and metastasis in breast cancer. Finally, these results of Oncomine analysis, GEPIA analysis and UALCAN analysis showing higher HCK expression in cancer tissues than that in non-cancer tissues were confirmed in fresh cancer tissues. The ratio of HCK expression in metastatic tumor was also higher than that in primary tumor. Collectively, these findings support that HCK is an adverse prognosis factor of breast cancer. In addition, we also found that HCK may affect progression of breast cancer by some immune-related biological pathways and processes.

These findings were consistent to other research on the role of HCK in cancer. Researchers have reported that HCK was an independent adverse prognostic factor for CRC patients and was associated with reduced chemosensitivity and acquired resistance in breast cancer 39,40. Activated membrane SFKs member, FGR and HCK can work in parallel to promote cancer development and weaken lymphocytic infiltration. Poh et al found that the inhibition of HCK expression can suppress myeloid cell-mediated colon cancer progression. The change of myeloid-related HCK expression owing to STAT3 activation can regulate cancer-associated macrophage polarization and the growth of colon cancer. Furthermore, inhibition of HCK expression can reduce the cancer burden in the mice model. They also found that high HCK expression is related to poor survival outcomes in CRC patients 16. Dong Wook Je et al found that HCK inhibitors can prevent proliferation and induce cell cycle arrest in pancreatic cancer cells. HCK knockdown can also inhibit proliferation and migration of pancreatic cancer cells 41. However, other studies found that in increased HCK expression in chronic myeloid leukemia (CML) and renal cancer is associated with an increased survival time 42,37. These findings suggest that the cancer origin and microenvironment may be important in the role of certain factors in cancer behavior. Other researchers also found *HCK* gene amplification and overexpression in gastric cancer 17 and colorectal cell lines 43. Furthermore, HCK overexpression may be caused by suppression of C-terminal Src kinase and Cbp/PAG regulation pathway, such as in hepatocellular carcinoma 44, and by tyrosine phosphatases, such as in breast cancer 45. TEL/ABL oncogenic fusion protein expression has been found in CML 46. TEL/ABL oncogenic fusion protein can activate ERK, AKT and other related pathways to promote the progression of tumor 47. This indicates that HCK may be an important regulator of TEL/ABL-dependent cancer progression because kinase-dead *HCK* mutants inhibited the progression of TEL/ABL transformed cells and the phosphorylation of ERK1/2 and AKT 48. This finding also shows that HCK can regulate the ERK/AKT pathway to depend cancer growth. Overall, most *in vitro* and *in vivo* animal experiments showed findings consistent to our study, that is, HCK negatively impacts survival outcomes and thus may as a potential therapeutic target for breast cancer treatment.

While the research may be the first research to evaluate the clinical implications of HCK expression in breast cancer patients, it also has some limitations. Firstly, the study was retrospective by design, conducted in a single center, and included a small sample size, thus limiting the generalizability of our conclusions. Studies that include more patients are therefore needed to explore the exact influence of HCK expression in breast cancer. Second, we only explore the regulatory pathway of HCK based on bioinformation tools and did not validate the molecular mechanisms using *in vitro* cell experiments and *in vivo* animal experiments to explain the biological function of HCK expression in breast cancer. Thus, future studies should aim to focus on biochemical experiments that could explain the mechanisms by HCK expression can affect the development of breast cancer and determine the feasibility of HCK-targeted drugs in breast cancer treatment.

Overall, this research found that HCK expression was higher in cancer tissues than that in non-cancer tissues and was related to distant metastasis and death. Ultimately, HCK is an adverse independent predictor of survival outcomes in breast cancer and may serve as a potential therapeutic target for breast cancer patients.

## Supplementary Material

Supplementary figures and tables.

## Figures and Tables

**Table 1:** Correlations between HCK expression and clinicopathological characteristics

Variables	HCK expression (%)	No HCK expression (%)	*P*-value
No. of Patients	47 (54.0)	40 (46.0)	
**Age (year)**			0.847
≤45	15 (31.9)	12 (30.0)	
>45	32 (68.1)	28 (70.0)	
**Other disease**			0.498
No	36 (76.6)	33 (82.5)	
Yes	11 (23.4)	7 (17.5)	
**Histological grade**			0.588
I	15 (31.9)	10 (25.0)	
II	26 (55.3)	22 (55.0)	
III	6 (12.8)	8 (20.0)	
**Tumor size (cm)**			0.008
Median (range)	1.69 (1.00-8.00)	0.75 (0.50-3.50)	
**No. of PALNs**			0.006
Median (range)	3.0 (0-27)	1.68 (0-17)	
**Menopausal status**			0.498
Premenopausal	26 (55.3)	25 (62.5)	
Postmenopausal	21 (44.7)	15 (37.5)	
**ER Status**			0.702
Positive	36 (76.6)	32 (80.0)	
Negative	11 (23.4)	8 (20.0)	
**PR Status**			0.487
Positive	36 (76.6)	28 (70.0)	
Negative	11 (23.4)	12 (30.0)	
**HER2 Status**			0.543
Positive	17 (36.2)	12 (30.0)	
Negative	30 (63.8)	28 (70.0)	
**Ki67 Status**			0.121
>20%	23 (48.9)	13 (32.5)	
≤20%	24 (51.1)	27 (57.5)	

No. of PALNs: number of positive axillary lymph nodes.

**Table 2:** Relationships between HCK expression and prognosis

Variables	HCK expression (%)	No HCK expression (%)	*P-*value
No. of Patients	47 (54.0)	40 (46.0)	
**Distant Metastasis**			0.002
Yes	26 (55.3)	9 (22.5)	
No	21 (44.7)	31 (77.5)	
**DFS (month)**			<0.001
Median (range)	88.34 (30-132)	115.38 (40-149)	
**Death**			<0.001
Yes	21 (44.7)	4 (10.0)	
No	26 (55.3)	36 (90.0)	
**OS (month)**			0.001
Median (range)	112.17 (41-143)	130.28 (59-149)	

**Table 3:** Univariate and multivariate cox regression analyses of clinicopathological factors for disease-free survival among these patients

Variables	DFS			
	Univariate analysis		Multivariate analysis	
	HR (95%CI)	*P*-value	HR (95%CI)	*P*-value
Age (year)	1.054 (0.524-2.119)	0.883	NA	
Other disease	1.262 (0.524-3.041)	0.604	NA	
**Histological grade**			NA	
I		0.312		
II	1.887 (0.806-4.420)	0.144		
III	1.974 (0.662-5.887)	0.223		
Tumor size (cm)	1.398 (1.182-1.655)	<0.001	2.558 (1.155-5.662)	0.021
No. of PALNs	1.029 (0.969-1.092)	0.357	NA	
Menopausal status	1.631 (0.840-3.168)	0.149	NA	
ER Status	1.300 (0.568-2.979)	0.535	NA	
PR Status	1.339 (0.608-2.949)	0.468	NA	
HER2 Status	1.092 (0.543-2.196)	0.804	NA	
Ki67 Status	1.199 (0.614-2.343)	0.596	NA	
HCK expression	3.225 (1.508-6.895)	0.003	1.293 (1.082-1.544)	0.005

NA: Non-analysis.

**Table 4:** Univariate and multivariate cox regression analyses of clinicopathological factors for overall survival among these patients

Variables	OS			
	Univariate analysis		Multivariate analysis	
	HR (95%CI)	*P*-value	HR (95%CI)	*P*-value
Age (year)	1.518 (0.682-3.381)	0.307	NA	
Other disease	1.317 (0.523-3.319)	0.559	NA	
**Histological grade**			NA	
I		0.878		
II	1.268 (0.491-3.279)	0.624		
III	1.278 (0.360-4.539)	0.704		
Tumor size (cm)	1.513 (1.187-1.929)	0.001	1.606 (1.245-2.072)	<0.001
No. of PALNs	1.022 (0.946-1.104)	0.580	NA	
Menopausal status	1.066 (0.484-2.348)	0.874	NA	
ER Status	1.274 (0.532-3.051)	0.587	NA	
PR Status	1.060 (0.440-2.554)	0.897	NA	
HER2 Status	1.830 (0.729-4.595)	0.198	NA	
Ki67 Status	3.292 (1.448-7.489)	0.004	3.413 (1.423-8.184)	0.006
HCK expression	5.707 (1.954-16.663)	0.001	4.725 (1.585-14.085)	0.005

NA: Non-analysis.
