# Supplementary material for: HCK can serve as novel prognostic biomarker and therapeutic target for Breast Cancer patients
Source: Int J Med Sci. 2020 Sep 30;17(17):2773–89. doi: 10.7150/ijms.43161 (PMC7645343; doi:10.7150/ijms.43161)
Supplement: Supplementary file 1 — Supplementary figures and tables. [file ijmsv17p2773s1.pdf]

Supplementary Figure 1 Coexpedia analysis of network comprising *HCK* and neighboring genes.

Supplementary figure 1

A

B

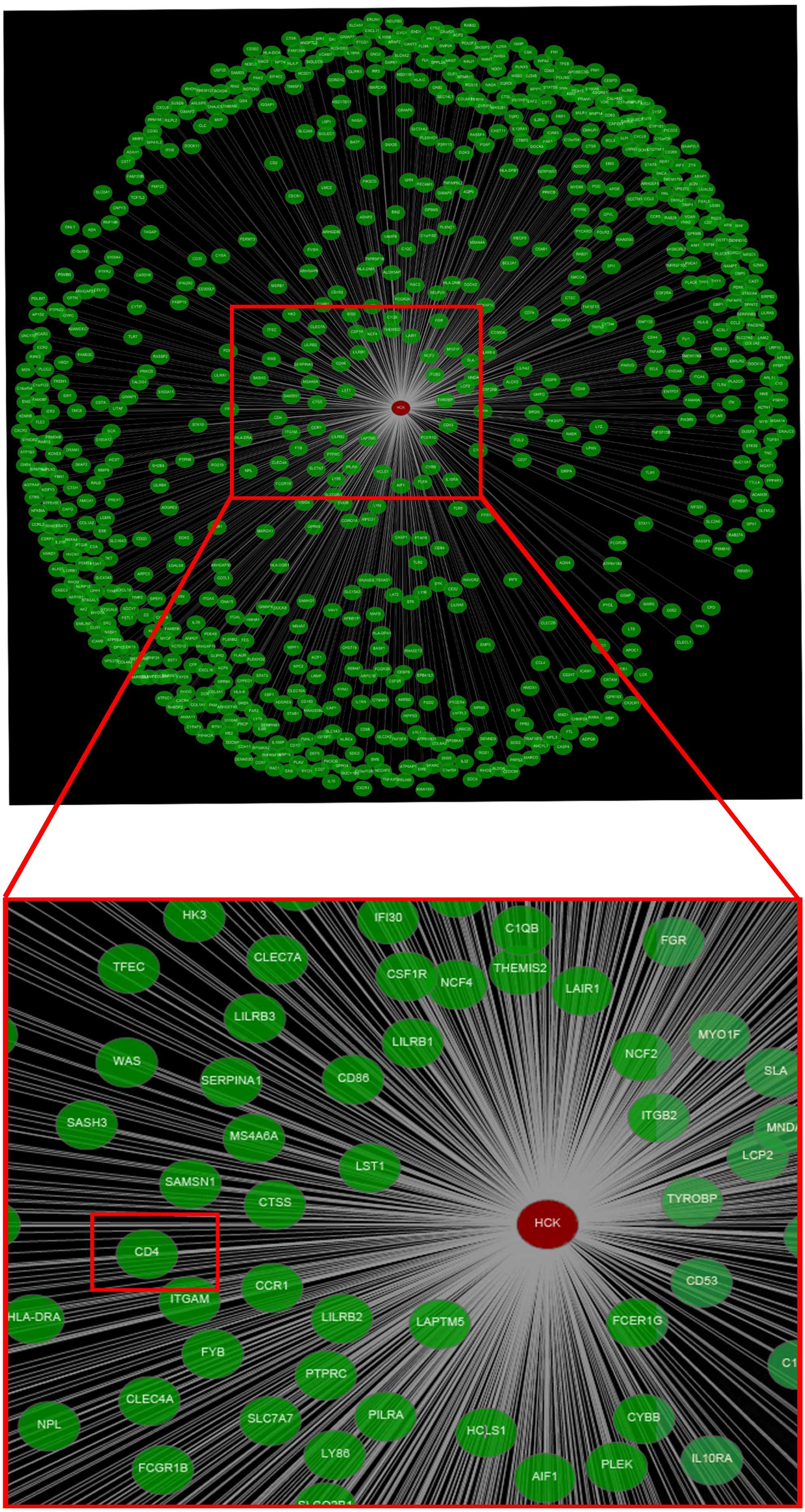

Supplementary table 1. The correlation analysis of HCK expression and pHCK expression in breast cancer patients

	pHCK expression				
HCK expression	Negative	Positive	Number	R value	*P*-value
Negative	30	10	40	0.327	0.002
Positive	20	27	47		
Number	50	37	87		
